# Self-Organized Near-Zero-Lag Synchronization Induced by Spike-Timing Dependent Plasticity in Cortical Populations

**DOI:** 10.1371/journal.pone.0140504

**Published:** 2015-10-16

**Authors:** Fernanda S. Matias, Pedro V. Carelli, Claudio R. Mirasso, Mauro Copelli

**Affiliations:** 1 Instituto de Física, Universidade Federal de Alagoas, Maceió AL 57072-970, Brazil; 2 Departamento de Física, Universidade Federal de Pernambuco, Recife PE 50670-901, Brazil; 3 Instituto de Física Interdisciplinar y Sistemas Complejos, IFISC CSIC-UIB, Campus Universitat de les Illes Balears, E-07122 Palma de Mallorca, Spain; Cuban Neuroscience Center, CUBA

## Abstract

Several cognitive tasks related to learning and memory exhibit synchronization of macroscopic cortical areas together with synaptic plasticity at neuronal level. Therefore, there is a growing effort among computational neuroscientists to understand the underlying mechanisms relating synchrony and plasticity in the brain. Here we numerically study the interplay between spike-timing dependent plasticity (STDP) and anticipated synchronization (AS). AS emerges when a dominant flux of information from one area to another is accompanied by a negative time lag (or phase). This means that the receiver region pulses before the sender does. In this paper we study the interplay between different synchronization regimes and STDP at the level of three-neuron microcircuits as well as cortical populations. We show that STDP can promote auto-organized zero-lag synchronization in unidirectionally coupled neuronal populations. We also find synchronization regimes with negative phase difference (AS) that are stable against plasticity. Finally, we show that the interplay between negative phase difference and STDP provides limited synaptic weight distribution without the need of imposing artificial boundaries.

## Introduction

Synchronization by neuronal oscillation is a ubiquitous phenomenon in the brain [1]. It has been reported in many species and in a variety of sensory and motor tasks [2, 3]. In particular, coherent oscillations in the cortex have been related to associative learning as well as working and long-term memory [4–6]. In these synchronized regimes, it is possible to determine the relative phase difference in the activation of the involved areas. In unidirectionally coupled motifs, for example, the relative phase difference is usually positive, which means that the sender population transfers information and activates the receiver population. However, it has been shown that, in the presence of dynamical inhibitory loops in the receiver population, unidirectionally coupled neuronal models may exhibit either positive or negative phase differences [7, 8]. This sender-receiver model [8] has been proposed as a mechanism to explain the reported mismatch between directional influence and negative time delay among regions of the monkey cortex [6, 8, 9].

The counterintuitive situation in which the receiver leads the sender, characterized by a negative phase difference, is called anticipated synchronization (AS) [10–12]. AS can be a stable solution of a sender-receiver (or, equivalently, a master-slave) system, if the receiver is also subject to a negative delayed self-feedback [13–21]. AS has been theoretically and experimentally studied in a number of physical systems [22–29]. Nonetheless, there are few investigation of AS in biological systems [30] as well as in the relation between anticipatory behavior and AS dynamics [31, 32]. In fact, the first observation of AS in a neuronal model was done by Ciszak et al [30]. It was shown that two unidirectionally coupled FitzHugh-Nagumo neuron models driven by white noise can exhibit AS in the presence of a negative delayed self-feedback in the receiver neuron. AS was also verified in other master-slave neuron models due to synaptic delays [33] and depolarization parameters [34]. A biologically plausible model for AS in neuronal systems was studied in [7], using three Hodgkin-Huxley neuron models coupled in a sender-receiver configuration, with the delayed self-feedback replaced by a synaptic loop mediated by an inhibitory neuron [7].

More recently, AS was numerically found between unidirectionally coupled neuronal populations in the presence of dynamical inhibitory loops [8]. It was argued that the reported positive Granger causality and negative phase difference among cortical areas [6, 9] could be the first experimental evidence of AS in the brain [8]. In a nutshell, it was experimentally observed that a brain region A could Granger-cause synchronized activity in a region B, yet the phase difference between the two could be negative [9]. This paradox was only apparent because, as the AS phenomenon clearly shows, temporal differences are not a good proxy for causality. Even a simple neuronal population model could reproduce time differences, coherence and GC spectra of the experimental data [8]. Additionally, it was shown that the time delay (or phase difference) between the master and the slave in the model is a smooth function of the excitatory and inhibitory weights. This means that the synaptic weights mediate transitions from positive time delay, called delayed synchronization (DS) regime, to negative time delay (AS regime).

These results, together with the experimental evidence that the synaptic weights can undergo spike-timing dependent plasticity (STDP) [35–37], open new perspectives in the study of neuronal network dynamics. In fact, the mechanisms relating synchronization and plasticity are still under investigation [38–43]. Based on STDP rules, the time difference between the spikes of pre- and post-synaptic neurons engenders modifications in the synaptic weights. On the one hand, synaptic changes induced by STDP rules can promote a transition between synchronized regimes. On the other hand, a given synchronization regime can strongly influence the relative spike times, and hence the STDP dynamics. Here, we investigate the interplay between STDP and the AS-DS transition. In other words, we study how STDP rules and the synchronization regimes work synergistically to determine the network dynamics.

Initially we study a simple 3-neuron motif which exhibits AS and DS regimes [7]. In the DS regime the master (pre-synaptic) neuron fires a spike before the slave (post-synaptic) neuron, which under STDP rules facilitates long term potentiation (LTP) [36, 44, 45]. In the AS regime the slave neuron fires a spike before the master neuron, contributing to long term depression (LTD). In turn, the increase (or decrease) of the excitatory synaptic weights via LTP (or LTD) generates modifications in the time delay. These successive interactions regulate the functional organization of a simple 3-neuron motif.

Next, we study the effect of STDP in the excitatory synapses between two unidirectionally coupled cortical-like populations which exhibits AS or DS. We investigate how the STDP rules applied at the neuronal level can influence synchronization at the populational level. We show that the interplay between these two different scales gives rise to emergent properties when compared with the 3-neuron motif. First, STDP and the inhibitory loop provide a new mechanism for near-zero-lag synchronization between distant cortical areas. Second, the AS regime is robust against STDP. Third, the transition from AS to DS can be controlled by the local amount of inhibition in the receiver population. We also show that, together with AS, STDP rules provide synaptic weight distributions that are stable, diverse and limited [46]. Furthermore, in the AS regime, such distributions are comparable to those observed experimentally in the cortex [47–49].

## Results

### Three-neuron motif

**Fig 1:**
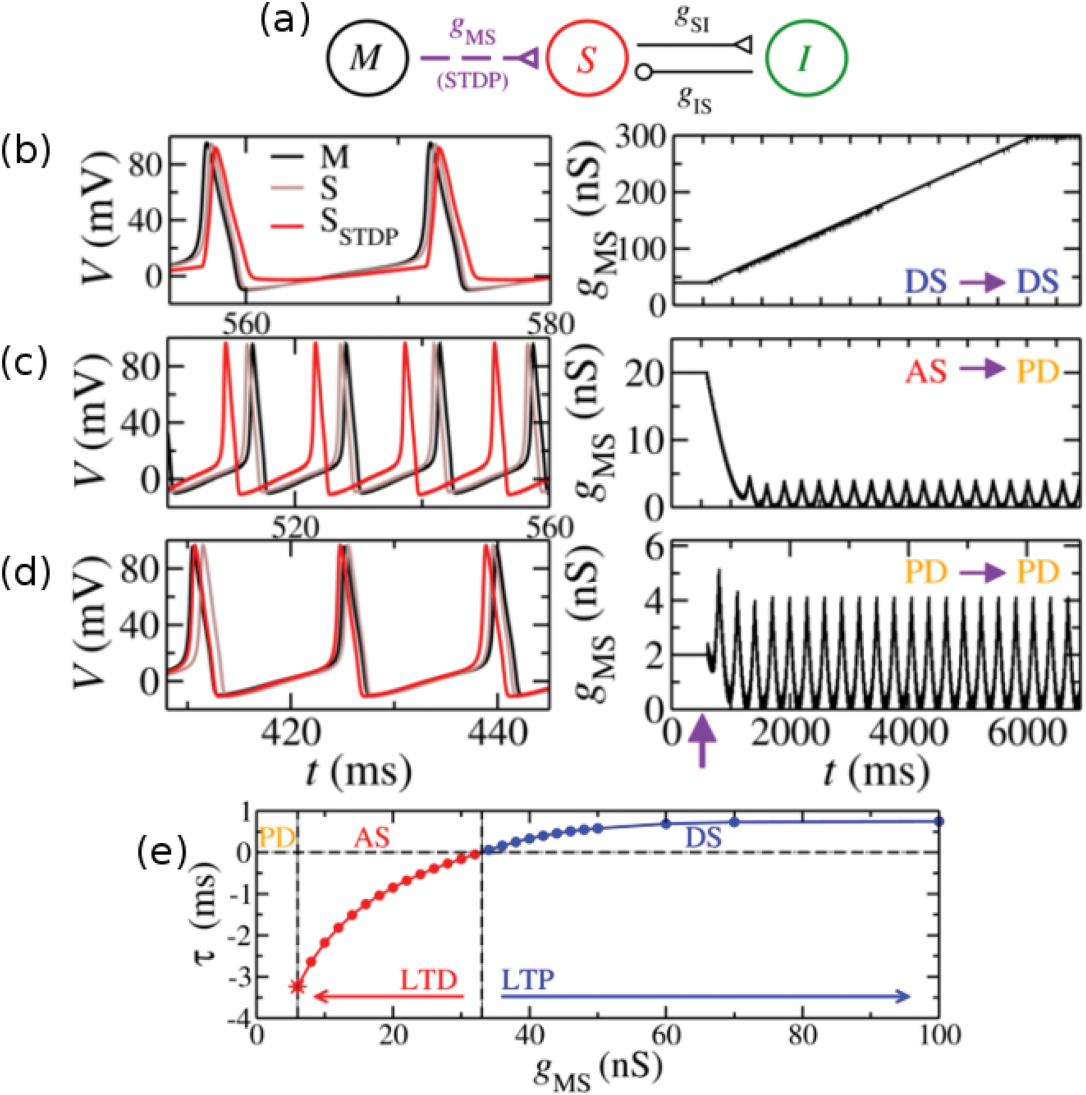
STDP in a microcircuit of three neurons. (a) Three neurons coupled by chemical synapses in the master-slave-interneuron (MSI) configuration. The excitatory synaptic weight *g*_*MS*_ may change through STDP rules. (b)-(d) Left: membrane potential of the master (M, black), the slave with no plasticity (S, grey) and the slave with STDP (S_STDP_, red). Right: The synaptic weight *g*_*MS*_ as a function of time. The vertical arrow indicates the moment at which plasticity rules were switched on. The system can exhibit synchronized regimes with positive time delay (delayed synchronization, DS) or negative time delay (anticipated synchronization, AS) and a phase-drift (PD) regime. (b) The system initiates in the DS regime and remains in it. The synaptic weight increases from the initial value to the upper boundary. (c) The system initiates in the AS regime, then *g*_*MS*_ decreases to values smaller than 6 nS and the phase-drift (PD) regime is reached. (d) The system initiates in the PD regime and ends in a different PD regime. (e) Without STDP, the time delay *τ* between M and S is a smooth function of the weight *g*_*MS*_. If we turn on STDP, the DS region (*g*_*MS*_ > 32 nS) leads to LTP whereas the AS region (6 < *g*_*MS*_ < 32 nS) leads to LTD. For *g*_*MS*_ < 6 nS the system exhibits a PD regime.

The simplest biophysical neuronal model which exhibits AS is the 3-neuron motif shown in Fig 1(a) [7]. It consists of three identical Hodgkin-Huxley neurons spiking periodically: the master (M), the slave (S), and the interneuron (I), coupled by chemical synapses. They are connected via two excitatory synapses, from M to S (*g*_*MS*_) and from S to I (*g*_*SI*_), as well as an inhibitory synapse from I to S (*g*_*IS*_, see Methods for more details). The time delay *τ* = *t* ^*S*^ − *t* ^*M*^ is defined as the spike timing difference between the S and M neurons. It was shown that without plasticity, this motif may present two phase-locking regimes: DS (*τ* > 0) and AS (*τ* < 0), as well as a phase-drift (PD) regime [7]. The membrane potentials of M and S shown in Fig 1(b)–1(d)) illustrate each regime.

In what follows, we apply STDP rules in the excitatory synapse from M (pre-synaptic neuron) to S (post-synaptic neuron). Thus, the excitatory synaptic conductance *g*_*MS*_ changes according to the spike timing difference between M and S following Eq 10. Typically, for unidirectionally coupled neurons, the post-synaptic neuron fires after the pre-synaptic neuron (characterizing a DS regime) promoting LTP. However, due to the existence of the AS regime in the 3-neuron motif, the presence of STDP can also cause a decrease of the synaptic weight (LTD).

The effect of STDP in the DS regime causes the synaptic weight *g*_*MS*_ to increase via LTP until the imposed upper boundary is reached (see right panel of Fig 1(b)). Although one could expect that larger values of the excitatory synaptic weight would facilitate near-zero-lag synchronization, in our model *τ* is a monotonically increasing function of *g*_*MS*_ (see Fig 1(e)). Therefore, STDP leads to an increase in *τ* (compare S and S_STDP_ in Fig 1(b)).

In the AS regime the post-pre order of spikes facilitates LTD. When we turn on STDP, the synaptic weight decreases. The final regime depends on the lower boundary *g* ^*min*^ of the algorithm. If *g* ^*min*^ < 6 nS, the system reaches a PD regime (compare S and S_STDP_ in Fig 1(c)). In the AS regime, both M and S oscillate with a period *T*_*M*_ = 14.7 ms, whereas in the PD regime the new period of the slave is 14.1 ms (i.e. it is faster than the master). After a transient time, the synaptic weight *g*_*MS*_ oscillates between 0 and 4 nS (see right panel of Fig 1(c)). On the contrary, if we choose 6 < *g* ^*min*^ < 32 nS, *g*_*MS*_ decreases until it reaches the boundary and the system remains in the AS regime. This means that AS is only stable for appropriated boundaries.

Finally, we applied STDP in a PD regime (see Fig 1(d)). Without STDP, the period of the slave is 14.5 ms. After the plasticity is switched on, the system reaches a different PD regime. The slave period is 14.1 ms and the synaptic weight *g*_*MS*_ oscillates between 0 and 4 nS. The oscillation period of *g*_*MS*_ is *T* ≃ 294 ms which is almost 20 times the neuron period and it is related to the STDP time scales.

In the absence of STDP, the time delay *τ* between M and S is a smooth function of *g*_*MS*_ [7], which is shown in Fig 1(e). Note that, as mentioned, larger values of *g*_*MS*_ provide larger *τ*. The three different situations reported above were obtained by selecting different initial values of the synaptic weight: *g*_*MS*_ = 40 nS (DS), *g*_*MS*_ = 20 nS (AS) and *g*_*MS*_ = 2 nS (PD). As expected, when we apply STDP rules in the AS regime the synaptic weight *g*_*MS*_ decreases by LTD, while in a DS regime, *g*_*MS*_ increases by LTP (see arrows in Fig 1(e)). If the system remains in a phase-locking regime (due to the boundaries), the final time-delay *τ* is determined by the final weight. In a nutshell, the presence of STDP in the 3-neuron motif takes the system close to the imposed boundaries of the STDP and far from the zero-lag synchronization solution.

### Neuronal populations

#### Synchronization is robust against STPD

**Fig 2:**
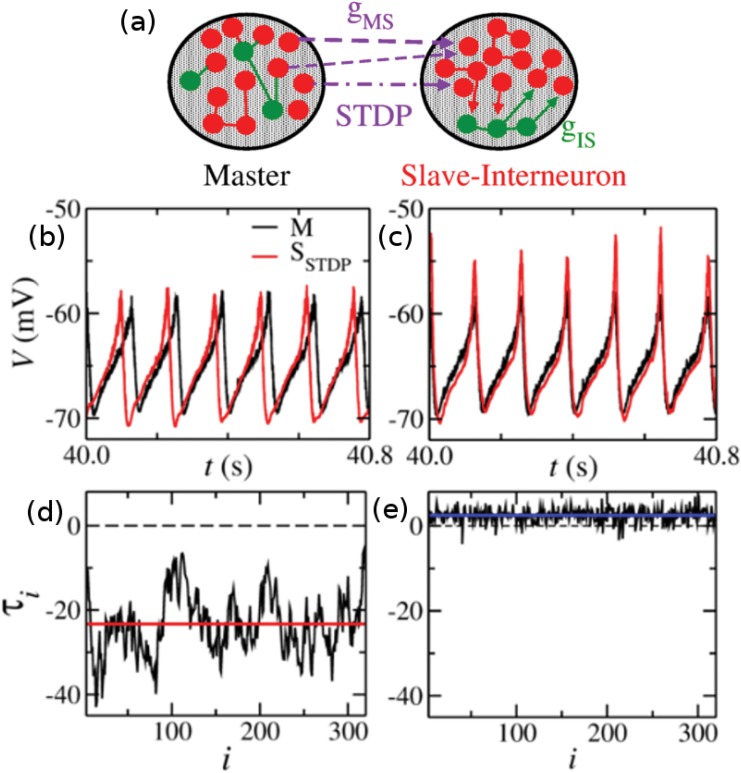
The interplay between phase difference and STDP in neuronal populations. (a) Master and Slave-Interneuron cortical-like populations. Each synaptic weight *g*_*MS*_ (from Master to the Slave) is subject to STDP rules. (b)-(c) Membrane potentials of M (black) and S (red) populations in the AS regime (b) and in the DS regime (c) in the presence of plasticity. (d)-(e) The time delay in each cycle *τ*_*i*_. The mean time delay *τ* (flat line) is negative in the AS regime (d) and positive in the DS regime (e). The AS and DS regimes are obtained modifying only one parameter in the model. The inhibitory conductances in the Slave-Interneuron population is set to *g*_*IS*_ = 4 nS in the AS regime (b) and (d), whereas *g*_*IS*_ = 16 nS in the DS regime (c) and (e).

In order to extend our results to a cortical-like region, we investigate the effects of STDP in a population model which can exhibit AS [8]. We numerically studied the synchronization properties of two populations composed of hundred of neurons, described by the Izhikevich model, unidirectionally coupled in a master-slave configuration (see Fig 2(a) and Methods for more details). Neurons from the Master (M) population project excitatory synapses (each one with synaptic weight *g*_*MS*_) to excitatory neurons from Slave-Interneuron (SI) population. The inhibitory loop that is mediated by the interneuron in the 3-neuron motif, is mediated by the inhibitory neurons inside the SI population. Thus, we assume that the slave population is composed by two subgroups: the excitatory neurons, called the Slave (S) sub-population, and the inhibitory neurons, called the Interneuron (I) sub-population. Each inhibitory synapse from I to S has synaptic weight *g*_*IS*_. Without plasticity rules, the populations can oscillate with a well defined mean period [8]. Moreover, their activity can synchronize and the mean time delay *τ* between the M and S populations can be positive (DS) or negative (AS, see Methods). Similarly to the 3-neuron motif, *τ* is a continuous and smooth function of the synaptic weights *g*_*MS*_ and *g*_*IS*_.

Here we investigate the effects of STDP only in the synapses from M to S allowing each synaptic weight *g*_*MS*_ to change according to the plasticity rules (see Eq 11 in the Methods section). After a transient time, the system reaches a synchronized regime. The mean membrane potential of the M and S populations are illustrated in Fig 2(b) and 2(c) for the AS and DS regimes respectively. In the AS regime the Slave precedes the Master, whereas in DS the Slave lags behind the Master. Due to the external noise and neuronal variability, the time delay in each period *τ*_*i*_ fluctuates around its mean value *τ* (see Fig 2(d) and 2(e) and Methods for more details).

**Fig 3:**
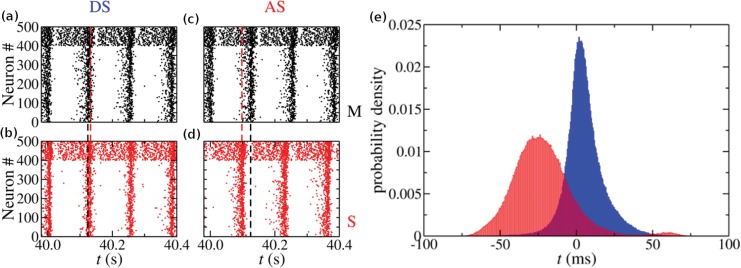
Neuronal firing patterns in the DS and AS regimes due to STDP. (a)-(d) Raster plots of each population in the DS and AS regimes. Black dots are neurons from the Master population. Red dots are neurons from the Slave-Interneuron population. Neurons 0 to 399 are excitatory, whereas neurons 400 to 499 are inhibitory. (e) Histogram of the time delay between the closest spikes in each cycle of all coupled pairs whose pre-synaptic neuron is in the M population and the post-synaptic neuron is in the S population. The blue distribution represents a DS regime and the red distribution represents an AS regime. The inhibitory conductances in the S population is set to *g*_*IS*_ = 16 nS in the DS regime, and *g*_*IS*_ = 4 nS in the AS regime.

Although synchronized oscillations are collective phenomena, the DS and AS regimes can also be represented at the neuronal level. The raster plots in Fig 3(a)–3(d) illustrate the oscillatory behavior of the coupled populations. Black (red) dashed lines indicate the time of peak average activity of the Master (Slave-Interneuron) population (see Methods for more details). In the DS regime the darker regions in the Slave-Interneuron population occur shortly after the ones in the Master population (see Fig 3(a) and 3(b)). On the contrary, in the AS regime, the darker regions in the Slave-Interneuron population occur before the ones in the Master population (see Fig 3(c) and 3(d)). However, there is a fraction of the neurons in the Master population which spikes before the peak of the Slave-Interneuron activity. The histograms in Fig 3(e) show the probability density of spike-timing intervals between each spike from neurons in the S population and the nearest spike from their respective pre-synaptic neurons in the M population. Although there are positive and negative values for the spike-timing intervals in both regimes, the peak and the mean of the distribution have positive values in the DS regime (blue, *g*_*IS*_ = 16 nS) and negative values in the AS regime (red, *g*_*IS*_ = 4 nS).

#### STDP promotes near-zero-lag synchronization

**Fig 4:**
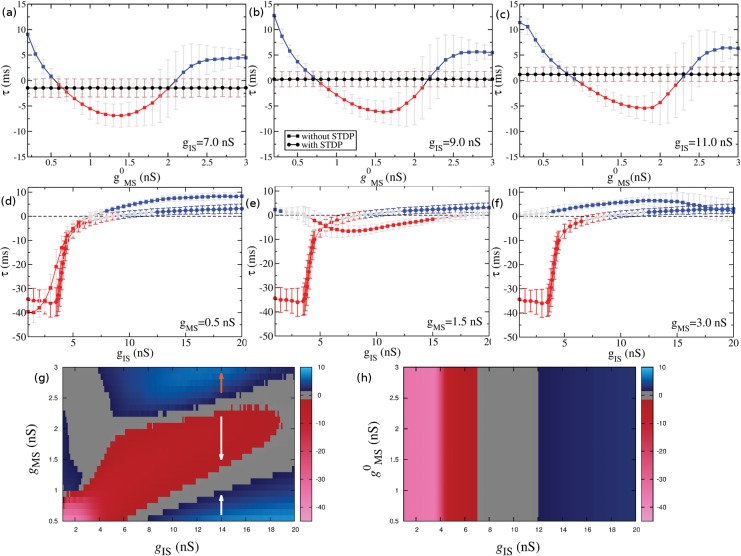
STDP changes the relation between the time delay *τ* and the synaptic weights. STDP promotes zero-lag synchronization (*τ* ≈ 0): (a)-(c) *τ* as a function of the initial excitatory weights $g_{MS}^{0}$ with and without STDP (represented by circles and squares, respectively). (d)-(f) *τ* as a function of the inhibitory weights *g*_*IS*_ with and without STDP. (g)-(h) *τ* (color coded) in the (*g*_*IS*_, $g_{MS}^{0}$) parameter space without plasticity (g) and with STDP (h). The near-zero-lag regime is represented by the grey regions, AS (*τ* < 0) by the red regions and DS (*τ* > 0) by the blue ones. In the presence of STDP, *τ* is determined by the local amount of inhibition *g*_*IS*_.

The presence of plasticity and the inhibitory dynamical loop can lead unidirectionally coupled neuronal populations to self-organize into near-zero-lag oscillations. The continuous transition from DS to AS, mediated by the excitatory synaptic weights *g*_*MS*_ in the absence of STDP, collapses into a flat line in the presence of STDP (see Fig 4(a)–4(c)). For 7 nS < *g*_*IS*_ < 12 nS the system exhibits time delay *τ* ≃ 0 (see Fig 4(h)). This means that independently of the initial values of the excitatory weights, the inhibitory loop together with STDP rules are sufficient to provide a *τ* ≈ 0 synchronized solution.

**Fig 5:**
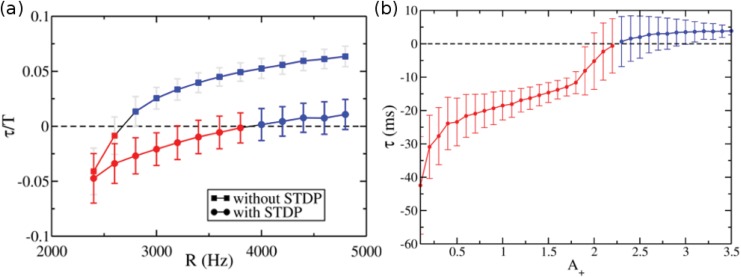
Robustness of synchronization regimes against model parameters. (a) Effect of external noise with and without plasticity: time delay normalized to the period of the Master population *τ*/*T* versus the Poisson rate *R* of the external noise. The inhibitory conductances in the Slave-Interneuron population is *g*_*IS*_ = 5 nS and *A*_+_ = 0.5. (b) Time delay *τ* versus the parameter of the STDP rule *A*_+_ (see Eq 11) for *g*_*IS*_ = 4 nS. A smooth transition from AS to DS can be observed.

Despite the time decay of the chemical synapses, the neuronal variability and the external noise, the network exhibits *τ* ≈ 0 synchronized solution for a large set of parameters. For large enough Poisson rate (*R* > 3500 Hz in our simulations, see Methods) the STDP rule brings the system closer to zero-lag synchronization (see Fig 5(a)). Although the conductance value of each individual synapse *g*_*MS*_ can change in time, the near-zero-lag regime is stable. In fact, in the near-zero-lag regime, the standard deviation of *τ* (≈ 1.5 ms, see Fig 4(a)–(c)) is much smaller than the mean period of each population (≈ 130 ms, see Fig 2(b) and 2(c)). Altogether, these results reveal a new mechanism which may contribute to the large-scale synchronization phenomena in the brain.

Interestingly, the result of the interplay between STDP and the collective oscillations cannot, in general, be predicted by analyzing the phase diagram without plasticity (Fig 4(g)). Assuming that all the synapses could be roughly described by a mean value, one could consider, for instance, the region 7 nS≲ *gIS* ≲ 16 nS (white arrows in Fig 4(g)). For low initial values of *g*_*MS*_, below the zero-lag transition region, the system is in the DS regime, in principle leading STDP to increase the excitatory synaptic conductances. For larger, intermediate initial values of *g*_*MS*_, the system is in the AS regime, which would lead STDP to reduce excitatory synaptic weights. Both conditions would push the system toward the zero-lag region, as is indeed observed in Fig 4(h) (although for a narrower interval of *g*_*IS*_ values). However, for even larger values of *g*_*MS*_ the system is again in the DS regime (Fig 4, orange arrow). Applying the same logic, one would expect STDP to increase *g*_*MS*_ even further, pushing the system away from the zero-lag regime. This is not verified in the simulations, suggesting that the actual STDP rule, which acts separately in each synapse, promotes a more robust synchronized regime than the one that would be reached if all synapses were identical.

#### Anticipated synchronization as an emergent property

In Fig 4(d)–4(f) we show the relationship between *τ* and the inhibitory weights *g*_*IS*_ with and without plasticity in the excitatory conductance *g*_*MS*_. In the presence of STDP, the time delay resists to change for inhibitory weights *g*_*IS*_ < 4 nS. However, when increasing *g*_*IS*_ beyond 4 nS, *τ* rapidly increases to near-zero values. Unlike the 3-neuron situation, in the cortical-like networks it is possible to start in a DS regime (without plasticity) and reach an AS regime via STDP or vice versa. The model also predicts that it is possible to start in an AS regime (or in a DS regime) and remains in AS (or DS) when STDP is turned on. In Fig 4(g) and 4(h) we compare the values of *τ* along the parameter space (*g*_*IS*_, $g_{MS}^{0}$) with and without plasticity. Without STDP, the combination of *g*_*MS*_ and *g*_*IS*_ that provides positive or negative *τ* is non trivial as shown in Fig 4(g). This means that STDP acts as an organizing mechanism for the network. For fixed STDP parameters there is a well-defined and continuous region of negative, positive and near-zero time delay synchronized solutions (see Fig 4(h)). Furthermore, with and without STDP, the time delay can be modulated by the amount of external noise, which could switch on or off the anticipation (see Fig 5(a)) Interestingly, the presence of plasticity allows the AS regime to persist for larger noise values and be robust to the parameters tat govern the STDP.

The transition from AS to DS is continuous and smooth when changing the parameters *A*_+_ and *A*_−_ in Eq 11. Fig 5(b) shows the relationship between *τ* and *A*_+_ for fixed *A*_−_ = 1.0. In fact, our model not only illustrates an example of AS in the presence of STDP but also shows that STDP enlarges the parameter region in which the time delay is negative.

#### STDP and negative phase difference stabilize synaptic weight distribution

**Fig 6:**
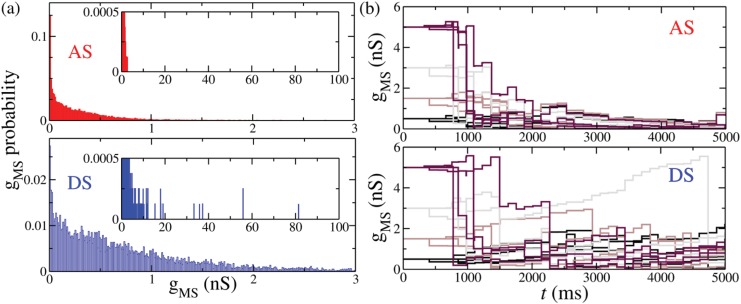
Synaptic weight distributions in the presence of STDP rules. (a) Histogram of the *g*_*MS*_ values. Inset: AS provides limited weight distribution even without an upper boundary. However, in the DS regime some synapses can grow unlimited. (b) Time evolution of four randomly chosen synaptic weights starting from different initial conditions in the AS and DS regimes. Each color represents a different simulation in which all initial synaptic conductances $g_{MS}^{0}$ were the same. The inhibitory conductances in the Slave-Interneuron population is *g*_*IS*_ = 4 nS in the AS regime, while *g*_*IS*_ = 16 nS in the DS regime.

A remarkable outcome of our model is related to the synaptic weight distribution when the system reaches an AS regime via STDP. As a result of the dynamical interaction between AS and STDP, the weight distribution obeys the three key properties required for biophysical reliability [46] as shown in Fig 6(a). First, the shape of the distribution is stable. Although each synapse can individually change in time, the distribution of all synaptic weights maintains a similar pattern along time. Second, it is diverse; it does not concentrate all the values at the boundaries. Third, it is limited i.e. there are no infinitely large synapses. More interestingly, synaptic weights do not explode even without an arbitrarily chosen boundary.

On the contrary, for a DS regime, the third property is not completely satisfied. Eventually it is necessary to choose an upper boundary for the weights, otherwise some of them grow beyond biophysical limits. In Fig 6(a) we see that there is a probability of finding arbitrarily large values of *g*_*MS*_ in the DS regime in the absence of a boundary, which does not happen for AS regime. However, the stability of the weights’ distribution and diversity of weights still occur. In Fig 6(b) we see the evolution of four randomly chosen synaptic weights starting from different initial conditions in the AS and DS regimes. In the DS regime there is a probability of extremely fast growing of the synaptic weights. On the contrary, in the AS regime all weights converge to small values. It is worth mentioning that these results do not depend on the initial values of *g*_*MS*_.

## Discussion

It is known that depending on the relative spike timing, synaptic weights can be modified through STDP mechanisms [37]. Moreover, previous works have shown how synaptic changes in the coupling of neural systems can impact their synchronization regimes, inverting the time lag [7, 8]. In the present paper we have investigated how the interplay between STDP and the synchronization regimes dynamically control the network function and connectivity.

In the population model, STDP acts at the synaptic level between each pair of pre-post synaptic neurons, whereas the synchronization time *τ* between the two populations is influenced by all *g*_*MS*_ values, reflecting a collective behavior. Therefore, the presence of plasticity could, in principle, hamper the synchronization. However the synergetic interplay between STDP and the AS-DS transition provides robust and stable synchronized regimes. We have shown that the AS regime is stable in a large set of parameter values in the presence of STDP rules. Moreover, due to the STDP the AS-DS transition is independent of the initial values of the excitatory weights. Consequently, the time delay can be controlled by the local amount of inhibition at the receiver population. For instance, if the Master population projects unidirectional synapses to two distinct slave populations, the phase difference between the two uncoupled slave population would depend on the strength in the local inhibitory pool.

### Master-Slave populations self-organizes into near-zero-lag synchronization

Zero-lag synchronization has been extensively studied in the brain. It was first reported in cat visual cortex [50], but has been widely found in neuronal functions, ranging from perceptual integration to the execution of motor behaviors [3, 51–54]. From the modeling viewpoint, several mechanisms have been pointed out as partially responsible for the enhancement of such synchrony. Among many, we can cite inhibitory synapses and gap junctions [55, 56], a canonical circuit of excitatory and inhibitory neurons for the hippocampal neurons [57–59] and synaptic plasticity mechanisms [60].

More recently, a robust mechanism, named dynamical relaying, has been proposed [61, 62] and tested in the thalamo-cortical [63] and hippocampal [64] circuits. The model proposes that two spatially separated areas can synchronize at zero lag if they are mutually coupled through a third area placed between them. The mechanism was found to be robust for certain parameters range, in the presence of inhomogeneity [62, 65], for modified motifs [66] and against STDP rules [39, 59, 67]. Here we show that STDP rules can also promote zero-lag synchronization in unidirectionally coupled motifs. Our results provide a new motif, different from those proposed in the dynamical relaying scenario, which allows near-zero-lag synchronization between distant cortical areas. Furthermore, we show that the zero-lag regime is stable, robust and can be controlled by the inhibitory synapses in the receiver region.

### Plasticity yields robust weight distributions and synchronized regimes

There have been several attempts to answer the question of how weight distributions and synaptic plasticity rules are related [45, 47, 48, 68, 69]. Typically, the experimental synaptic weight distributions reported in the literature have similar shapes: a monotonic decay function with maximum probability near zero, but different scales [47]. In addition, plenty of studies (in the cortex, hippocampus, and cerebellum) strongly suggest the existence of a large majority of undetectable synapses with almost zero weight. In our model, AS can shape the weight distributions between the Master and Slave populations into biophysical plausible patterns [47]. Such distributions present a monotonic decay and obey three key properties [46]: they are stable, diverse and limited (see Fig 6(a)).

Furthermore, a negative time delay could facilitate the responsiveness of post-synaptic neurons. For example, the olfactory receptor neurons (ORNs) and the projection neurons (PNs) of male moths are unidirectionally coupled. Interestingly, it has been verified that the response time of the PNs is smaller than the one of the ORNs [70]. Rospars et al. [70] have suggested that this shorter latency of PNs could be explained by AS. Since the PNs exhibit a multiphasic response pattern with an initial excitation followed by an inhibition, they could be subject to a similar dynamical inhibitory loop as the one required for AS.

Several works support that communication between brain regions is more efficient at near zero-lag synchronization [53, 54, 71]. However, an explanation for the underlying mechanisms and the functional significance of the reported phase differences between cortical regions [6, 9, 72–75] is still lacking. Although it is often assumed that the phase difference between distant areas reflects the transmission time of neural activity [73, 74, 76], it has been shown that, for the same given directional influence, the phase difference can be positive, negative or near zero [6, 8, 9]. Here we have shown that STDP does not destroy these synchronization regimes, but rather makes them more robust, and, in principle, tunable by local inhibition.

## Methods

### The master-slave-interneuron microcircuit

The typical master-slave configuration consists of two neurons unidirectionally coupled by an excitatory synapse. Here we have studied the 3-neuron motif illustrated in Fig 1. The circuit is composed by a master-slave coupling and a dynamical inhibitory loop mediated by an interneuron [7]. Each neuron is described by a Hodgkin-Huxley model [77], which consists of four coupled ordinary differential equations associated to the membrane potential *V* and the ionic currents flowing across the axonal membrane corresponding to the Na^+^, K^+^ and leakage currents. The gating variables for sodium are *h* and *m* and for the potassium is *n*. The equations are [78]:

$$\begin{array}{ccc} C_{m}\frac{dV}{dt} & = & {\bar{G}}_{Na}m^{3}h\left(E_{Na}-V\right)+{\bar{G}}_{K}n^{4}\left(E_{K}-V\right)\\ & & +G_{m}\left(V_{rest}-V\right)+I+\sum I_{syn} \end{array}$$ (1)

$$\begin{array}{ccc} \frac{dx}{dt} & = & \alpha_{x}\left(V\right)\left(1-x\right)-\beta_{x}\left(V\right)x\;, \end{array}$$ (2)

where *x* ∈ {*h*, *m*, *n*}, *C*_*m*_ = 9*π* *μ*F is the membrane capacitance of a 30×30×*π* *μ*m^2^ equipotential patch of membrane [78] and ∑*I*_*syn*_ accounts for the synapses from other neurons. The external constant current *I* = 280 pA settles the period of the neuron as *T* = 14.7 ms for ∑*I*_*syn*_ = 0. The reversal potentials are *E*_*Na*_ = 115 mV, *E*_*K*_ = −12 mV and *V*_*rest*_ = 10.6 mV, with maximal conductances ${\bar{G}}_{Na}=1080\pi$ mS, ${\bar{G}}_{K}=324\pi$ mS and *G*_*m*_ = 2.7*π* mS, respectively.

The excitatory and inhibitory synapses are modulated by AMPA (A) and GABA_A_ (G). The fraction *r* ^(*i*)^ (*i* = *A*,*G*) of bound synaptic receptors is modeled by:

$$\begin{array}{c} \frac{dr^{\left(i\right)}}{dt}=\alpha_{i}\left[T\right]\left(1-r^{\left(i\right)}\right)-\beta_{i}r^{\left(i\right)}, \end{array}$$ (3)

The values of the rate constants are *α*_*A*_ = 1.1 mM^−1^ms^−1^, *β*_*A*_ = 0.19 ms^−1^, *α*_*G*_ = 5.0 mM^−1^ms^−1^, and *β*_*G*_ = 0.30 ms^−1^. [*T*] is the neurotransmitter concentration in the synaptic cleft, given by:

$$\begin{array}{c} \left[T\right]\left(V_{pre}\right)=\frac{T_{max}}{1+e^{\left[-\left(V_{pre}-V_{p}\right)/K_{p}\right]}}, \end{array}$$ (4)

where *T*_*max*_ = 1 mM^−1^, *K*_*p*_ = 5 mV and *V*_*p*_ = 62 mV. The synaptic current at a given synapse is given by

$$\begin{array}{c} I^{\left(i\right)}=g_{i}r^{\left(i\right)}\left(E_{i}-V\right), \end{array}$$ (5)

where *V* is the postsynaptic potential, *E*_*A*_ = 60 mV and *E*_*G*_ = −20 mV are the reversal potentials. The maximal weight *g*_*i*_ in the excitatory synapse from M to S (called *g*_*MS*_) and in the inhibitory synapse from I to S (called *g*_*IS*_) are the important parameters of our model. Particularly, *g*_*MS*_ can be modified by STDP rules, as explained below. The existence of an AS regime specially depends on *g*_*IS*_ [7]. Here we use *g*_*IS*_ = 40 nS. The excitatory synaptic weight from S to I is fixed *g*_*SI*_ = 40 nS, but our results are almost independent of its specific value. We use fourth order Runge-Kutta’s method for numerical integrations with a time step of 0.01 ms. Simulations were performed using a C++ code which is available upon request.

### Modeling cortical-like neuronal populations

The two unidirectionally coupled neuronal networks in Fig 2 might represent cortical regions in the brain [62]. Each population is composed of hundreds of neurons described by the Izhikevich model [79] given by:

$$\begin{array}{c} \frac{dv}{dt}=0.04v^{2}+5v+140-u+\sum I_{x}, \end{array}$$ (6)

$$\begin{array}{c} \frac{du}{dt}=a\left(bv-u\right). \end{array}$$ (7)

where *v* is the membrane potential and *u* the recovery variable, which account for activation of K^+^ and inactivation of Na^+^ ionic currents. The currents in ∑*I*_*x*_ represent the synaptic currents. If *v* ≥ 30mV, then *v* is reset to *c* and *u* to *u* + *d*. For each excitatory neuron: (*a*, *b*) = (0.02, 0.2) and (*c*, *d*) = (−65, 8) + (15, −6)*σ* ^2^, whereas for each inhibitory neuron: (*a*, *b*) = (0.02, 0.25) + (0.08, −0.05)*σ* and (*c*, *d*) = (−65, 2). *σ* is a random variable uniformly distributed on the interval [0, 1] which determines the proportion of different neurons (regular spiking, intrinsically bursting, chattering—including type I and II excitability).

The excitatory (AMPA) and inhibitory (GABA_A_) synaptic current are mediated by:

$$\begin{array}{c} I_{x}=g_{x}r_{x}\left(E_{x}-v\right), \end{array}$$ (8)

where *x* = *A*,*G*, *E*_*A*_ = 0 mV and *E*_*G*_ = −65 mV. Unless otherwise stated all excitatory (inhibitory) weights are set to *g*_*A*_ = 0.5 ms (*g*_*G*_ = 4 nS). The dynamics of the fraction of bound synaptic receptors *r*_*x*_ is given by:

$$\begin{array}{c} \tau_{x}\frac{dr_{x}}{dt}=-r_{x}+\sum_{k}\delta \left(t-t_{k}\right). \end{array}$$ (9)

The summation over *k* stands for pre-synaptic neurons. The time decays are *τ*_*A*_ = 5.26 ms and *τ*_*G*_ = 5.6 ms. Each neuron receives an independent Poisson input, representing *n* pre-synaptic neurons spiking with rate *R*/*n*. The noise mimics excitatory chemical synapses from other brain regions. The rate *R* strongly influences the main oscillation frequency of each population. We use Euler’s method with a time step of 0.05 ms to integrate the differential equations.

The Master (M) population is composed of 500 neurons (80% excitatory, 20% inhibitory), each one receiving 50 synapses (sparse connectivity ≈ 10%) from randomly selected neighbors (excitatory or inhibitory) in the same population. For an external rate *R* = 2400 Hz, the mean membrane potential *V* of this population oscillates with a mean period *T*_*M*_ ≈ 130 ms, corresponding to a frequency *f* ≈ 7.7 Hz, which is related to theta oscillations reported in several experiments [53, 74, 75].

The Slave-Interneuron population is also composed of 500 neurons. In order to maintain the analogy with the 3-neuron motif we called Slave (S) population the sub-group of 400 excitatory neurons and Interneuron (I) population the sub-group of 100 inhibitory neurons belonging to the cortical-like Slave-Interneuron network. Each excitatory neuron from the S population receives 40 excitatory synapses from neighbor neurons belonging to S, 20 synapses from excitatory neurons from the M population (*g*_*MS*_, which characterizes the master-slave coupling) and 10 synapses from the interneurons in the I population (with conductances *g*_*IS*_, which play the role of the delayed self-feedback responsible for AS). Only the excitatory synaptic weights *g*_*MS*_ evolve under STDP rules.

Each neuron from the I population receives 10 inhibitory synapses from randomly selected neighbors neurons belonging to the same I population and 40 excitatory synapses from randomly selected neurons belonging to the S population. Results are quantitatively similar if we measure the mean membrane potential of just the S population or the S and I populations together. In fact, for all set of parameters employed here, S and I are synchronized. Then, the S and I populations can be considered as sub-populations of the same cortical region or well separated regions in the brain oscillating with the same frequency.

We use a sliding window (from 5 to 8 ms) to decrease the noise effect and to determine the time of the membrane peak in each period $t_{i}^{x}$ (*x* = M,S,I index the population and *i* the period). In all calculations we discount a transient time of 10 s and run the simulation until *t* = 50 s. The period of each population in each cycle is given by $T_{i}^{x}\equiv t_{i+1}^{x}-t_{i}^{x}$ and we calculate the mean period *T*_*x*_ and its variance. Similarly, the time delay in each cycle is defined as $\tau_{i}=t_{i}^{S}-t_{i}^{M}$. Then we obtain the mean time delay *τ* and its standard deviation, which is plotted as the error bars in Figs 4 and 5.

### STDP between unidirected coupled networks

In the 3-neuron motif the *g*_*MS*_ synaptic weight evolves under the additive rule:

$$\begin{array}{c} \Delta g=\{\begin{array}{cc} A_{+}\text{exp}\left(-t/\tau_{+}\right), & \text{if }t>0\\ -A_{-}\text{exp}\left(t/\tau_{-}\right), & \text{if }t<0, \end{array} \end{array}$$ (10)

with *A*_+_ = *A*_−_ = 1 nS, *τ*_+_ = *τ*_−_ = 10 ms and artificial boundaries 0 < *g*_*MS*_ < 300 nS.

For the populations, each excitatory synaptic weight *g*_*MS*_ from the Master to the Slave is subject to the following hybrid rule [80]:

$$\begin{array}{c} \Delta g=\{\begin{array}{cc} A_{+}\exp\left(-t/\tau_{+}\right), & \text{if}\;t>0\;\text{(additive}\;\text{LTP)}\;\\ -A_{-}g\exp\left(t/\tau_{-}\right), & \text{if}\;t<0\;\text{(multiplicative}\;\text{LTD)} \end{array} \end{array}$$ (11)

where *t* = *t* ^*S*^−*t* ^*M*^. It is worth mentioning that if we use an additive rule to LTD, it is necessary to choose a lower boundary for *g*_*MS*_ in order to avoid negative weights. Unless otherwise stated *τ*_+_ = *τ*_−_ = 5 ms, *A*_+_ = 0.5 nS, *A*_−_ = 1.0 and there are no arbitrarily chosen boundaries.
